# Herpes Simplex-1 and Toxoplasma gondii in Obsessive-Compulsive Disorder: clinical and brain imaging correlates

**DOI:** 10.1192/j.eurpsy.2022.1650

**Published:** 2022-09-01

**Authors:** A. Maia, J. De Souza Queiroz, B. Barahona-Correa, J. Oliveira, A.J. Oliveira-Maia

**Affiliations:** 1 Nova Medical School, Mental Health, Lisbon, Portugal; 2 Centro Hospitalar de Lisboa Ocidental, Mental Health And Psychiatry, Lisbon, Portugal; 3 Champalimaud Centre for the Unknown, Neuropsychiatry Unit, Lisbon, Portugal

**Keywords:** obsessive-compulsive disorder, herpes simplex 1, toxoplasma gondii, brain imaging

## Abstract

**Introduction:**

Obsessive-compulsive disorder (OCD) is a chronic, prevalent, and highly impairing psychiatric illness. While its aetiology remains unknown, several infectious agents have been associated to obsessive-compulsive symptoms, including herpes simplex virus 1 (HSV-1) and *Toxoplasma gondii.*

**Objectives:**

To evaluate the serostatus for HSV-1 and *Toxoplasma gondii* in sample of patients with OCD, as well as its clinical and brain imaging correlates.

**Methods:**

Twenty-six patients with OCD and 30 healthy controls recruited in Lisbon were assessed for sociodemographic and clinical characteristics using the Yale-Brown Obsessive-Compulsive Scale-II (YBOCS-II) and the Beck Depression Inventory-II (BDI-II). Seropositivity for HSV-1 and *Toxoplasma gondii* was assessed in serum using ELISA, and volumes of cortical and subcortical structures were assessed using T1-weighted magnetic resonance imaging.

**Results:**

YBOCS-II and BDI-II scores were significantly higher in patients, while age, sex, smoking status, and seropositivity for HSV-1 and *Toxoplasma gondii* were similar between the two groups. Among OCD patients, those seropositive for HSV-1 had significantly lower volumes of total white-matter, total grey-matter, left and right putamen, while for HSV-1 seropositive healthy controls, only the last two were significantly smaller. In multiple regression analyses to control for age, associations between HSV-1 and brain volumes were conserved, while the effect of age was not significant. No significant differences were found in brain volumes of patients with OCD according to seropositivity for *Toxoplasma gondii.*

**Conclusions:**

Our preliminary results suggest that in patients with OCD, seropositivity to HSV-1 is associated with smaller volumes of total white- and grey-matter in the brain.

**Disclosure:**

No significant relationships.

